# Toxin-Antitoxin Systems in Estuarine *Synechococcus* Strain CB0101 and Their Transcriptomic Responses to Environmental Stressors

**DOI:** 10.3389/fmicb.2017.01213

**Published:** 2017-07-06

**Authors:** David Marsan, Allen Place, Daniel Fucich, Feng Chen

**Affiliations:** Institute of Marine and Environmental Technology, University of Maryland Center for Environmental Science, BaltimoreMD, United States

**Keywords:** *Synechococcus*, toxin–antitoxin system, persister cell, stress response, *relB/relE*

## Abstract

Bacterial toxin–antitoxin (TA) systems are genetic elements composed of a toxin gene and its cognate antitoxin, with the ability to regulate growth. TA systems have not previously been reported in marine *Synechococcus* or *Prochlorococcus*. Here we report the finding of seven TA system pairs (Type II) in the estuarine *Synechococcus* CB0101, and their responses of these TA genes to under different stress conditions, which include; nitrogen and phosphate starvation, phage infection, zinc toxicity, and photo-oxidation. Database searches discovered that eight other marine *Synechococcus* strains also contain at least one TA pair but none were found in *Prochlorococcus*. We demonstrate that the *relB/relE* TA pair was active and resulted in RNA degradation when CB0101 was under oxidative stress caused by either zinc toxicity or high light intensities, but the growth inhibition was released when the stress was removed. Having TA systems allows *Synechococcus* CB0101 to adapt to the low light and highly variable environments in the Chesapeake Bay. We propose that TA systems could be more important for picocyanobacteria living in the freshwater and estuarine environments compared to those living in the open ocean.

## Introduction

Cyanobacteria are widely distributed in diverse habitats and they are able to adapt to variable and even extreme environments (Tandeau de Marsac and Houmard, 1993). Estuaries are among the most productive yet variable aquatic environments on Earth. Mixing of fresh and marine waters provides strong environmental gradients to the cyanobacteria living in these ecosystems. Estuarine cyanobacteria (e.g., *Synechococcus*) contribute significantly to global primary production and are at the interface of direct anthropogenic affects (Ray et al., 1989; Affronti and Marshall, 1993; Iriarte and Purdie, 1994). In estuaries, the bioavailability of nutrients (e.g., N and P), light intensity, and trace metals (e.g., Fe and Zn) are highly variable. Due to the complex geochemistry of these compounds and physical dynamics, understanding cyanobacterial response to the estuarine environment is important toward understanding the function of ecosystems.

Indeed, in their long evolutionary history, cyanobacteria have developed highly refined response strategies shared with other prokaryotes under various stressors (Schwarz and Forchhammer, 2005; Lewis, 2007). One common successful survival strategy for prokaryotes is the ability to undergo reversible growth arrest (Schwarz and Forchhammer, 2005; Lewis, 2007) using chromosomal toxin–antitoxin (TA) systems. TA systems have been documented in many organisms, especially *Escherichia coli*, to cope with various stresses by reducing growth, inhibiting growth or killing a subpopulation of cells (Kolodkin-Gal et al., 2009; Van Melderen and Saavedra De Bast, 2009; Yamaguchi et al., 2011). The freshwater filamentous cyanobacteria *Anabaena* sp. PCC7120 contains numerous chromosomal TA systems, some of which are predicted to promote survival under particular stresses (Ning et al., 2013).

Toxin–antitoxin systems, typically consist of an auto-regulated operon encoding a stable toxin and a labile antitoxin (Gerdes et al., 2005; Yamaguchi et al., 2011; Schuster and Bertram, 2013). TA systems have been grouped into types I, II, III, IV, and V classes based on the nature of antitoxin (Gerdes and Wagner, 2007; Fozo et al., 2008; Fineran et al., 2009). Antitoxins of type II, the most abundant type of TA, are proteins that inactivate toxins by forming TA complexes (Gerdes et al., 2005). The type II toxins are typically mRNA specific endoribonucleases or mRNA interferases (Yamaguchi et al., 2011). Binding to the promoter by a TA complex toxin neutralization by formation of a TA complex results in translation inhibition.

Type II systems have been found in plasmids and chromosomes of free-living bacteria (Pandey and Gerdes, 2005; Makarova et al., 2009; Leplae et al., 2011). It is believed that chromosomal TA systems enable bacteria to adapt to stressful environments (Gerdes et al., 2005; Yamaguchi et al., 2011). Stressful conditions trigger degradation of antitoxins by stress-induced proteases, and production of toxins from TA systems (Yamaguchi et al., 2011). Free toxins cause growth arrest or cell death of cells by inhibiting protein synthesis or DNA replication (Yamaguchi et al., 2011). The fact that possessing TA systems can help prokaryotic cells to become more resistant to stress has been supported by a series of recent experimental findings (Hazan et al., 2004; Prysak et al., 2009; Singletary et al., 2009; Christensen-Dalsgaard et al., 2010).

Despite the ubiquity of TA systems in other prokaryotic organisms, they have not previously been reported in marine picocyanobacteria such as *Synechococcus* or *Prochlorococcus*. Previous studies have hypothesized that a lack of TA systems in some prokaryotes is related to their small genome sizes (<3 Mb) and relatively simple life style in stable environments (Pandey and Gerdes, 2005). Currently, more than 60 marine picocyanobacterial genomes have been sequenced (NCBI search as of January 2016). In general, genome sizes of marine *Synechococcus* and *Prochlorococcus* are smaller than 3 Mb (Dufresne et al., 2008; Shi and Falkowski, 2008; Scanlan et al., 2009). Recently, we identified seven putative chromosomal TA pairs in the genome (2.8 Mb) of *Synechococcus* strain CB0101, which was isolated from the Chesapeake Bay (Marsan et al., 2014).

Marine *Synechococcus* can be divided into three major subclusters, 5.1, 5.2, and 5.3 (Dufresne et al., 2008; Scanlan et al., 2009; Huang et al., 2012b). Subclusters 5.1 and 5.3 *Synechococcus* are present in coastal and oceanic waters, while subcluster 5.2 *Synechococcus* mainly occupies estuaries (Chen et al., 2006). Compared to coastal and oceanic *Synechococcus*, much less is known about the ecological function and genomic evolution of estuarine *Synechococcus*. Subcluster 5.2 *Synechococcus* dominate the estuarine environment (Larsson et al., 2014) making up 20–40% of phytoplankton chlorophyll-a during the summer (Wang et al., 2011) and contain a novel pigment gene cluster not seen in other subclusters (Larsson et al., 2014). *Synechococcus* strain CB0101 (a member of subcluster 5.2) is able to grow in a wide range of salinities (0–30 ppt) and temperatures (4–32°C), and is often subjected to viral infection (Wang and Chen, 2008; Wang et al., 2011; Huang et al., 2012a). The presence of TA systems in the genome of CB0101 leads to the hypothesis that estuarine cyanobacteria similar to CB0101 retain TA systems to aid in adaptation to strong environmental gradients in the estuary.

In this study, we identified and characterized the chromosomal TA systems found in *Synechococcus* strain CB0101 (Marsan et al., 2014). The expression of TA systems in CB0101 in response to stressful environmental conditions (nutrient, metal toxicity, light intensity, and phage infection) were analyzed using RNA-Seq, confirmed through qPCR, and further confirmed through Western blotting of extracts. Time series experiments displayed the activation and shutdown of the toxin and antitoxin proteins when a stressful environment (i.e., Zn toxicity or photo-oxidative stress caused by high light intensity) was encountered and subsequently relieved. When CB0101 was under stress, it slowed down the growth rate, and meanwhile an increase in the ratio of toxin vs. antitoxin was observed. When the stressor is removed, growth rate increases and the toxin/antitoxin ratio decreases. Genomic searches led to the finding of TA systems in eight other marine *Synechococcus*. This is the first identification, characterization, and functional confirmation of TA systems in marine picocyanobacteria.

## Materials and Methods

### Cyanobacterial Strains and Stress Experiments

*Synechococcus* CB0101 is a well-referenced euryhaline strain isolated from the Chesapeake Bay, with a sequenced genome, and numerous cyanophages isolated from it. Cultures of CB0101 were grown in SN medium with 15 ppt salinity and vitamin B12 (10 μg/L) (referred to as SN15 medium hereafter) at 23°C under 15 μE m^-2^ s^-1^ continuous light. Filtered (0.2 μm pore size) air was bubbled into individual 500 mL cell culture flasks. For the RNA-Seq analysis, CB0101 cultures were initially grown under the above conditions and duplicates were subjected to four different conditions for 72 h: (1) Control, where the culture was grown in SN15 medium; (2) Nitrogen depleted condition, where the cultures were cultivated in the SN15 medium that did not contain sodium nitrate; (3) Phosphate deplete condition, where the cultures were grown in the SN15 medium that lacked dipotassium phosphate; (4) Zinc toxic condition, where the cultures were grown in the SN15 medium amended with 50 μM of zinc sulfate heptahydrate. The growth of cultures was monitored by counting cells using an Accuri C6 flow cytometer. The specific growth rate was calculated from the logarithmic change where X1 and X2 are densities at times t1 and t2. Samples were taken at 0 min and after 72 h, and mRNA was extracted immediately after sampling.

For the phage infection experiment the length of the lytic cycle was determined by one-step growth curve. Once the lytic cycle was determined, three time points were identified to correlate with attachment (30 min), latent (5 h), and lysis (12 h). Triplicate cultures of CB0101 were grown to exponential phase (10^8^ cells ml^-1^), and then infected with S-CBP1 (Wang and Chen, 2008) podovirus (10^8^ infective phage particles ml^-1^) for a multiplicity of infection (MOI) of 1. Control cultures were amended with sterilized SN15 medium. At the designated time points mRNA was extracted.

For the light intensity experiment, exponentially growing CB0101 culture (in SN15 medium) was split into five 25-ml cell culture flasks, which were exposed to five different light intensities (15, 50, 100, 150, and 200 μE m^-2^ s^-1^). The entire culture was extracted and mRNA and protein were separated for qPCR and Western blot analysis, respectively.

### Extraction of mRNA and qPCR Reaction

Samples were collected and immediately processed for mRNA analysis. RNA was separated using Trizol extraction. mRNA was extracted from the total RNA using Ambion MICROBExpress kit (Life technologies, AM1905), modified with four specific oligo primers for rRNA depletion of CB0101 ribosomes. rRNA removal evaluation was conducted using the Agilent 2100 Bioanalzyer resulting in 98% removal of rRNA and confirmed the presence of high quality RNA. The NEXTflex RNA-Seq kit (BIOO Scientific, #5129-01) was then used to prepare mRNA libraries for sequencing using the Illumina Hi-Seq. RNA libraries were created and barcodes assigned to each sample for multiplexing of a single Hi-Seq lane. RNA-Seq was carried out by the Institute of Genome Sciences (IGS) using a Hi-Seq with 100 bp-paired end read length. As a further confirmation, qPCR was used to validate the expression of each TA pair transcript expression.

### TA System Identification in CB0101 and Completely Sequenced *Synechococcus* and *Prochlorococcus* Genomes

BLASTCLUST (-L 0.75 –S1.0) searches for all identified TA genes grouped by family and cluster was used to identify TA genes within *Synechococcus* CB0101. These TA systems were further validated using the RASTA-Bacteria and TA finder programs (Sevin and Barloy-Hubler, 2007; Shao et al., 2011). Further, this method was used with representatives of each cluster used as queries in a BLAST search (*e*-value 0.01) against ∼60 completely sequenced *Synechococcus* and *Prochlorococcus* genomes available on the NCBI Microbial Genomes website at the time of this analysis (January 2016) (Federhen, 2015). Significant hits among proteins encoded in these genomes were classified as toxins or antitoxins; in the case of multiple matches to different TA families, the protein was assigned according to the highest-scoring match TA query. Co-directed genes with adjacent chromosome locations belonging to different toxin/antitoxin families were recorded as a TA pair.

### Western Analysis

Western blot analysis was conducted using a procedure described by Mahmood and Yang (2012). Amino acid sequences for all seven TA pairs were provided to GenScript for epitope analysis. Results were analyzed using their Optimum Antigen design tool and the *relB^2^/relE^1^* pair was selected for u production due to large transcript changes observed under each stress condition. Genscript synthesized each antigenic peptide (TARLPDDLTAELDAC and CVLVVRVGHRKEVYR for RelB*^2^* and RelE*^1^*, respectively) and added an additional cysteine residue to allow for conjugation to the KLH adjuvant. These were used for immunization of New Zealand white rabbits. Specific antibodies were isolated from the resulting serum by affinity purification using the synthesized peptide as bait. Antibodies were tested for reactivity by an ELISA assay with the peptide used to generate the antibody as the target coating the wells of a 96-well plate.

Western blot analysis was performed against samples from the resulting cultures using constant whole cell equivalents (∼2 × 10^7^ cells) mixed in running buffer were used for each Western. A one to 2000 dilution of the primary antibody was used. NuPAGE Novex gels with 4–12% Bis-tris gel with MES running buffer was used. Proteins were transferred to PVDF 0.2 μM membrane (Bio-Rad) using the Bolt Mini Blot Module (Life Technologies) and western blot carried out using the iBind Western Blot Apparatus (Life Technologies). Affinity-purified rabbit polyclonal antibodies specific to each *relB^2^/relE^1^* were used as the primary probe in western blotting. An anti-rabbit IgG (H&L) (GOAT) antibody that was peroxidase conjugated (Bio-Rad) was used as the secondary probe. Luciferase signal was visualized by incubation in Clarity Enhanced chemiluminescent (ECL) substrate (Bio-Rad) and imaged using a FluorChem E (Protein Simple). Quantification of band intensities was performed using Imageview software (Protein Simple).

## Results

### Annotated Toxin/Antitoxins in CB0101

**FIGURE 1:**
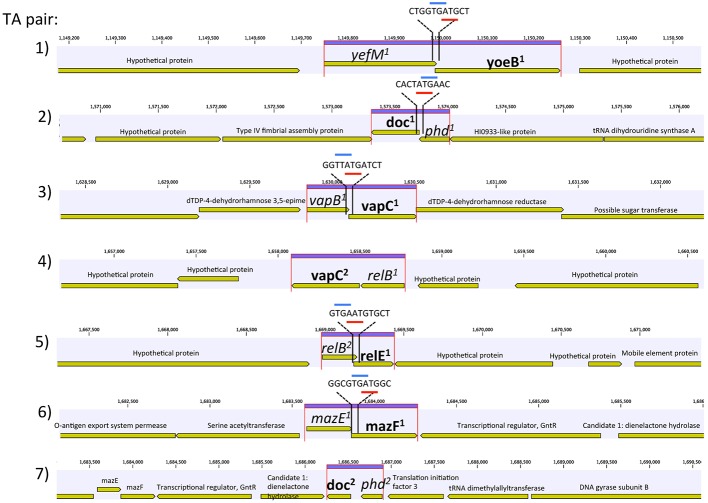
Genetic organization of the seven toxin–antitoxin (TA) systems of *Synechococcus* CB0101. Schematic representation of the seven TA pairs (highlighted in purple) and associated genes. Gene overlap is depicted by overlay with blue and red representing start and stop codons, respectively. Toxin is represented by bold and antitoxin by italics.

Genome annotation revealed the presence of seven chromosomal pairs of TAs, homologous to *E. coli* Type II (**Table 1** and **Figure 1**). Each TA pair was analyzed using InterProScan 5 and ExPASy for motif, PI, and molecular weight prediction (Supplementary Table S1). The type II TA pairs in CB0101 included *yefM^1^/yoeB^1^*, *mazE^1^/mazF^1^*, *relB^2^/relE^1^*, *vapB^1^/vapC^1^*, *relB^1^/vapC^1^*, *phd^1^/doc^1^*, and *phd^2^/doc^2^* all of which involve tight protein-to-protein interactions and translational arrest.

**Table 1:** The outcome of protein BLAST search of the seven *Synechococcus* CB0101 TA gene pairs.

TA Pair #	TA family	Closest neighbor	Identity	*E*-value
1	*yefM^1^*	*Synechococcus* sp. KORDI-49	76%	3E-38
1	**yoeB***^1^*	*Synechococcus* sp. WH8103	81%	1E-43
2	*phd^1^*	*Cyanobium* sp. CACIAM 14	87%	2E-36
2	**doc***^1^*	*Collimonas fungivorans*	50%	4E-33
3	*vapB^1^*	*Cyanobium* sp. PCC7001	75%	2E-33
3	**vapC***^1^*	*Cyanobium* sp. PCC7001	74%	7E-63
4	*relB^1^*	*Synechococcus* sp. WH5701	72%	1E-14
4	**vapC***^2^*	*Synechococcus* sp. WH5701	81%	4E-67
5	*relB^2^*	*Thioalkalivibrio sulfidiphilus*	55%	4E-19
5	**relE***^1^*	*Thioalkalivibrio sulfidiphilus*	64%	5E-28
6	*mazE^1^*	*Thauera phenylacetica*	59%	2E-24
6	**mazF***^1^*	*Polaromonas glacialis*	71%	5E-63
7	*phd^2^*	*Synechococcus* sp. CB0205	93%	0E+00
7	**doc***^2^*	*Cyanobium* sp. CACIAM 14	67%	5E-32

*Toxin is represented by bold and antitoxin by italics. All matches represent a best fit to length (>90%).*

The amino acid sequences of each TA pair from CB0101 were BLAST searched in GenBank to identify their closest homologs (Federhen, 2015). The closest neighbors to the TA pairs of CB0101’s are shown in **Table 1**. The DNA sequence identities of these closest hits varied from 50 to 93% among the 7 TA pairs. Two-thirds of TA genes were related to the TA genes in picocyanobacteria (mainly marine *Synechococcus* and *Cyanobium*), while one-third of CB0101 TA genes seem to be most similar to those in heterotrophic bacteria, i.e., *Collimonas fungivorans*, *Thioalkalivibrio sulfidiphilus*, and *Polaromonas glacialis* (**Table 1**). Interestingly, the TA systems were found in diverse ecotypes of marine *Synechococcus*, i.e., KORDI-49 (coastal), WH8103 (open ocean), WH5701 (estuarine), and CB0205 (estuarine). Most pairs exhibit highly basic and corresponding acidic PI values strengthening the case for their tight binding with each other (Supplementary Table S1). Many of the pairs contain one to three nucleotide open reading frame overlaps suggesting transcriptional coupling (**Figure 1**). The genome location of each TA pair falls within regions defined as genomic islands. Genomic islands (GIs) are large regions (more than eight kilobases long) of non-conserved, non-core genes that are sporadically distributed among the genome. Genomic islands were identified using the IslandViewer program.

### Responses of *Synechococcus* TA Systems to the Environmental Stresses

**FIGURE 2:**
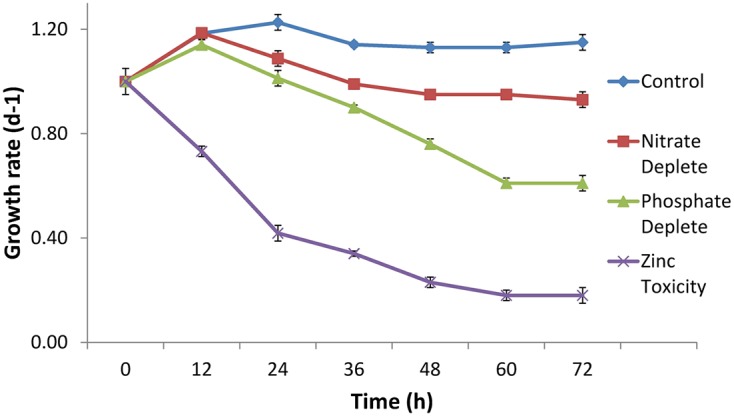
Growth rate of *Synechococcus* CB0101 during a 72 h period under different stress conditions: control medium, nitrogen limitation, phosphate limitation, and zinc toxicity (*n* = 3). See Chapter 3 for detailed explanation on *Synechococcus* CB0101 response to these conditions.

The growth rate of CB0101 under different stress conditions (nitrogen depletion, phosphorus depletion, and zinc toxicity) is shown in **Figure 2**. Under phosphate depletion and zinc toxicity growth was significantly slower than control (*p* < 0.05). High zinc exposure completely suppressed the growth of CB0101 (*t*-test value 2.7, *p* = 0.09).

RNA-Seq analysis was conducted to determine the transcriptional response of CB0101 to the four stress conditions (nitrate depletion, phosphorous depletion, zinc toxicity, and phage infection) (**Table 2** and Supplementary Table S2). The transcripts of toxin *relE^1^* were progressively upregulated 2.3-, 3.1-, and 10.6-fold (*p* < 0.01 and minimum 2-fold) in nitrate depletion, phosphate depletion, and zinc toxicity, respectively (TA pair #5, **Table 2**). *relE^1^* was also upregulated two fold at 30 min post infection of phage S-CBP1. The antitoxin gene *relB^2^* was downregulated by 2.5-fold when CB0101 was grown under nitrate depletion (TA pair #5, **Table 2**).

**Table 2:** Expression of seven *Synechococcus* CB0101 toxin/antitoxin pairs under various stress conditions detected by RNA-Seq and qPCR.

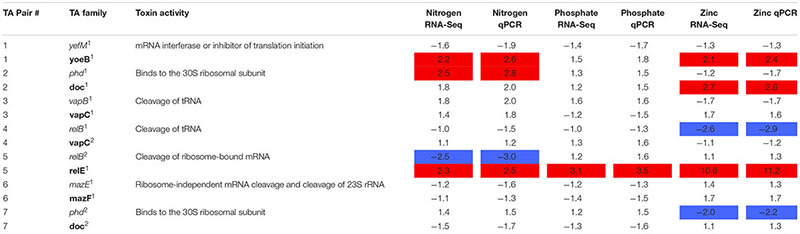

*Upregulation (red) or down regulation (blue) is based on expression of the control sample (p < 0.01 and minimum twofold). Toxin is represented by bold and antitoxin by italics*.

Other upregulated toxin genes included *doc^1^* (2.7-fold in zinc toxicity) and another *yoeB^1^* (2.2- and 2.1-fold in both nitrate depletion and zinc toxicity). The antitoxin *phd^1^* was upregulated 2.5-fold only under nitrate deplete conditions. *relB^2^* was only downregulated (2.5-fold) under nitrogen deplete conditions. While *relB^1^* was downregulated 2.6-fold under zinc toxic conditions.

In order to confirm the RNA-Seq expression data, PCR primers were made for each TA pair and TA gene transcript abundance was measured using qRT-PCR (**Table 2** and Supplementary Table S3). The same samples were used from the RNA-Seq experiments to verify results (*p* < 0.01 and minimum twofold). The qRT-PCR further confirmed the RNA-Seq data that the TA systems were active under the different stresses examined (**Table 2**). In general, the fold changes based on qRT-PCR were agreeable to those based on the RNA Seq results.

To demonstrate the TA systems were active at the protein level, we selected one TA pair (*relB^2^/relE^1^*) to further study using peptide specific antibodies. This TA pair was selected because the toxin was expressed at the transcript level, showed the largest expression difference and optimally designed antibodies could be made to both proteins. The nearly complete degradation of RNA (tRNAs, mRNA, and ribosomal RNAs) of CB0101 under high Zn conditions was observed, consistent with *relE* acting as an endoribonuclease to inhibit translation (Supplementary Figure S1). The bioanalyzer chip displays that the RNA of each condition other than the Zn experiment remained intact (Supplementary Figure S1).

### Production of *relE^1^* and Growth Decline of CB0101

**FIGURE 3:**
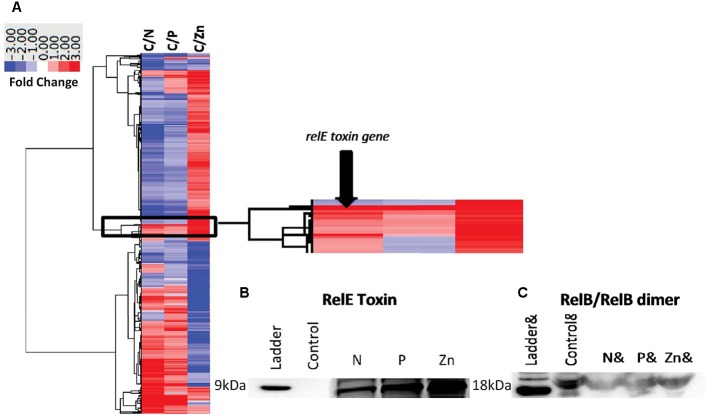
Global transcript expression of *Synechococcus* CB0101 to three stressors measured using RNA-Seq (*p* < 0.01 and minimum twofold) and compared to a control expression. **(A)** Heat map and clustering of differentially expressed genes. Stress conditions are all compared to Control, with C/N representing nitrogen depletion, C/P phosphate depletion, and C/Zn zinc toxicity representing 843 (27%), 368 (12%), and 1,195 (38%) differentially expressed genes identified from 3,173 total genes. Dark red equates to a strong increase (>3-fold), while dark blue to a strong decrease (>3-fold) in gene expression. Zoomed image of the expression from the toxin–antitoxin pair *relB^2^/relE^1^*. **(B)** Detection of the toxin RelE^1^ and **(C)** antitoxin dimer RelB^2^/RelB^2^ proteins by Western blot analysis from each stress condition including nitrogen (N) and phosphate (P) depletion, and zinc toxicity (Zn). Constant cell equivalents were used.

The *relE^1^* and *relB^2^* genes on CB0101’s chromosome encode RelE and RelB homologous to the *E. coli* RelE (24% identity) and RelB (41% identify), respectively. RelE is predicted to be an 84-residue sequence at 9.53 kDa, while RelB is predicted at 73 residues and 8.08 kDa (Supplementary Table S1). Expression of mRNA for the *relB^2^/relE^1^* pair in response to nitrate depletion, phosphate depletion and zinc toxicity are shown in **Figure 3A**. The mRNA expression of the *relB^2^/relE^1^* pair has been described above. The antibodies were used to confirm the presence of each protein (**Figures 3B,C**). The expressed RelB^2^/RelE^1^ proteins in all stress conditions were detected using specific antibodies loaded with constant cell equivalents (**Figures 3B,C**). Under the normal growth condition (control) a dimer of RelB^2^/RelB^2^ was detected at ∼18 kDa (**Figure 3C**), while the toxin protein RelE^1^ was not detected at its predicted weight 9 kDa (**Figure 3B**). When CB0101 became stressed in all three treatments, toxin protein RelE^1^ (band 9 kDa) became more prevalent and the dimer of antitoxin/antitoxin protein RelB^2^/RelB^2^ (band 18 kDa) declined (**Figures 3B,C**).

### Reversible *relB^2^*/*relE^1^* Expression When the Stress Is Removed

**FIGURE 4:**
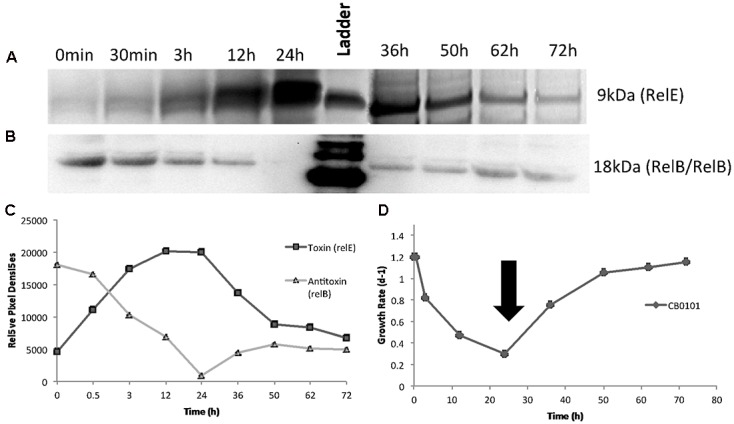
Time series detection of RelB^2^/RelE^1^ proteins by Western blot analysis during zinc toxicity and subsequent releasing at 30 h using constant whole cell equivalents (∼2 × 10^7^ cells) mixed in running buffer. NuPAGE Novex gels with 4–12% Bis-tris gel with MES running buffer was used. **(A)** Toxin RelE^1^; **(B)** Antitoxin dimer RelB^2^/RelB^2^; **(C)** Pixel density analysis to determine relative protein abundance; **(D)** Growth rate of *Synechococcus* CB0101 during the time series with black arrow representing media change (release of zinc toxic conditions) at 35.5 h.

After confirming the presence of both TA proteins (RelB^2^/RelE^1^) with western blots in nitrogen deplete, phosphate deplete and zinc toxic treatments, a time series experiment was set up to determine the progression of the TA pair when CB0101 was exposed to zinc toxicity (**Figure 4**). In this experiment, CB0101 was exposed to the same zinc toxicity (as the previous experiment), and subsamples were collected at nine different time points (0, 0.5, 3, 12, 24, 36, 38, 50, 62, and 72 h). CB0101 was grown in the presence of zinc for 36 h; the culture was then exposed to fresh non-toxic media and allowed to grow for another 36 h (**Figure 4**). When the zinc was added to the culture the toxin (RelE^1^) began to accumulate over time (**Figure 3A**; 0–24 h). Upon removal of zinc at 36 h, toxin protein began to dissipate gradually over time (**Figure 4A**; 36–72 h). The opposite trend was observed with the dimer of antitoxin/antitoxin. Upon exposure to zinc the RelB^2^/RelB^2^ complex decreased gradually and was fully degraded within 24 h (**Figure 4B**). When the culture was released from zinc toxicity at 36 h, the dimer of RelB^2^/RelB^2^ recovered almost instantaneously (**Figure 4B**). Western pixel densities show the inverse synchronization of the toxin and antitoxin proteins (**Figure 4C**). The growth rate of CB0101 was inhibited with zinc exposure in the first 24 h, and recovered after the release of zinc stress (**Figure 4D**).

### Light Response for *relB^2^*/*relE^1^*

**FIGURE 5:**
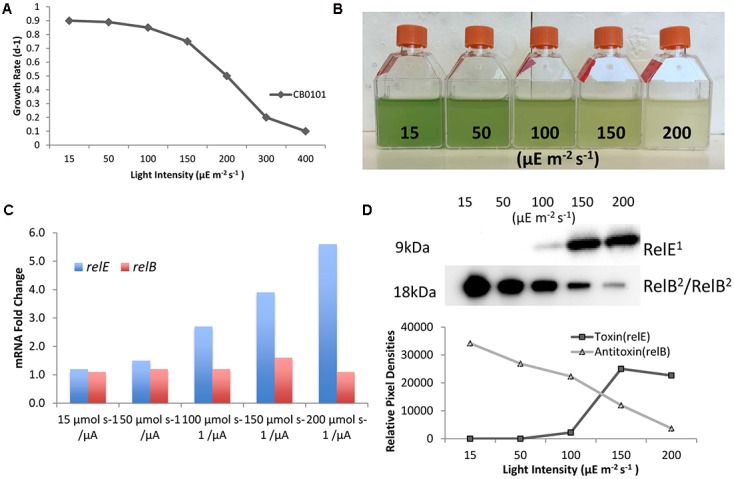
Photo-oxidative stress caused by different light intensities induces TA systems and arrests growth of CB0101. **(A)** Growth rate of *Synechococcus* CB0101 under different light intensities. **(B)** CB0101 cultures exposed to different light intensities 15, 50, 100, 150, and 200 μE m^-2^ s^-1^ for 48 h. **(C)** Expression of CB0101 *relB^2^/relE^1^* TA system responding to different light intensities detected by qPCR. **(D)** Western blot analysis of RelE^1^ toxin at 9 kDa and RelB^2^/RelB^2^ dimer at 18 kDa under different light intensities with pixel densities measured.

Since zinc toxicity is inducing *relB^2^/relE^1^* and this type of stress induces oxidative stress, we believe a better representation of niche adaptation would be responses to photo-oxidative stress. A major source of oxidative stress which photosynthetic organisms deal with is the production of reactive oxygen species (ROS) (Latifi et al., 2009) at high light intensities. CB0101 grew rapidly at low light intensities (15–50 μE m^-2^ s^-1^) and the growth was severely inhibited when light intensity exceeded 200 μE m^-2^ s^-1^ (**Figure 5A**). The level of photo bleaching increased with increasing light intensity (**Figure 5B**).

The upper Chesapeake Bay, in which CB0101 thrives in, is a low light environment due to increased particles input from the river. The qPCR analysis of each TA pair showed the expression of *relE^1^* was upregulated (2.7-, 3.9-, and 5.6-fold) when CB0101 was exposed to light at 100, 150, and 200 μE m^-2^ s^-1^, respectively (**Figure 5C**). The transcripts of two other toxins, *yoeB^1^* and *doc^1,2^*, were upregulated (2.4- and 2.6-fold, respectively) at the highest light intensity. Lastly, the mRNA of two antitoxins, *relB^1^* and *phd^2^* were downregulated at the highest light intensity. The Western blot of RelB^2^/RelB^2^ showed the presence of a dimer (18 kDa), which progressively decreased as the light intensity increased (**Figure 5D**). Conversely, the RelE^1^ toxin became visible at 100 μE m^-2^ s^-1^ and increased at 150 and 200 μE m^-2^ s^-1^.

## Discussion

### Why Do *Synechococcus* spp. Contain TA Systems?

The presence of seven putative pairs of TA systems in the chromosome of CB0101 is the first description in marine cyanobacteria (**Table 1** and **Figure 1**). The number and type of TA systems in CB0101 are comparable to other prokaryotes (Schuster and Bertram, 2013). TA systems have been found in prokaryotic plasmids and chromosomes. While the plasmid-encoded TAs are known to stabilize plasmids in cells, the chromosome TA systems have multiple functions (Christensen et al., 2001). Four possible mechanisms for the presence of TA systems in chromosomes have previously been identified: (1) TA systems on chromosomes may fulfill a similar function to plasmid types and mediate stabilization of important genetic regions (Leplae et al., 2011); (2) Protection against invading DNAs such as plasmids and phages (Christensen et al., 2001; Hazan et al., 2004); (3) Formation of bacterial persister cells (Lewis, 2010); (4) Regulation of biofilm formation or global regulators of translation (Kolodkin-Gal et al., 2009).

We believe that at least three of the four above-mentioned mechanisms are applicable to TA systems in CB0101. First, TA systems of CB0101 are in proximity to important genes such as Type IV pilus (+187,951 bp), DNA repair (-53,681 bp), rod shape-determining proteins (*Mre*) (+32,834 bp), SOS-response repressor and protease *LexA* (+7,498 bp) (mechanism 1). All of which are involved in functions to boost niche ecotype or overall survival. Secondly, the up-regulation of the *relE^1^* toxin of the *relB^2^*/*relE^1^* pair occurred within 30 min of cyanophage infection (Supplementary Table S2). Expression of *relE^1^* may cause the cell to arrest translation, thus slowing or stopping the phage from replicating (mechanism 2). Thirdly, TA systems may allow CB0101 to develop dormancy or persister cells in response to environmental stresses (mechanism 3). This is reflected by the possibility for CB0101 to arrest translation and growth during stressful environmental conditions by regulating its TA systems such as *relB^2^*/*relE^1^* (**Figure 4**). CB0101 was able to resume its growth when the stress was removed, likely undergoing a persister state (**Figure 4**). A bacterial persister state is a quasi-dormancy state where the cells may recover and proliferate if enough antitoxin is produced to neutralize the toxin (Christensen et al., 2001; Lewis, 2010). Formation of persister cells regulated by TA systems could be an important mechanism for *Synechococcus* species living in dynamic or unstable environments. Transformation of these *Synechococcus* CB0101 TA systems into *E. coli* is necessary to determine the relationship between transcriptional regulation of the TA systems and the change in growth rate.

### The *relB/relE* System

The expression of antitoxins and toxin genes, *relB^2^*/*relE^1^*, were tightly regulated by the various stress conditions (**Figures 4**, **5**). The mechanisms behind how *relB^2^/relE^1^* respond to stress could be homologous to other prokaryotic systems such as *E. coli* (Christensen et al., 2001). Typically, transcription of the TA operon is auto-regulated by binding of the antitoxin or by the TA system to the promoter (Bukowski et al., 2011). Depending on the stoichiometric ratio of antitoxin to toxin, several types of complexes may be formed with distinct affinities to the promoter (Li et al., 2008; Overgaard et al., 2008; Bøggild et al., 2012; Taylor et al., 2014). This coincides with the time-series experiment where the antitoxin RelB^1^ rapidly recovered after the stress was released (**Figure 4**).

Our studies show that growth inhibition of CB0101 could be completely eliminated by subsequently ending the stress condition, and a near instantaneous recovery ofRelB^2^ (**Figure 4**). The *relB^2^/relE^1^* TA pair was active and arrested translation when under zinc oxidative stress, and the arrest was reversible when the stress event was removed (**Figures 1**, **4**). This result is in accordance with the previous demonstration that the overproduction of TA toxin caused reversible growth arrest (Overgaard et al., 2008; Bøggild et al., 2012). When CB0101 was exposed to high Zinc toxicity over 24 h, the toxin production (RelE) increased gradually, while antitoxin RelB was degraded over time (**Figure 4**). Such a post-transcriptional regulation has been seen in *E. coli* where antitoxins are digested by stress-induced proteases. More toxins are produced when the TA operon is derepressed with the reduction of antitoxin (Yamaguchi and Inouye, 2011). Therefore, it is possible that the chromosomal *relB^2^*/*relE^1^* system of CB0101 represents a growth modulator that may induce a persister state to enhance fitness and competiveness under particular stress conditions, such as metal or oxidative stress. As a result, cyanobacteria with this TA system can persist for a long period of time under stressful conditions and revive when environmental stresses are removed.

### Ecological Significance and Oxidative Response of *relB/relE*

Oxidative stress caused by high light intensities is known to be one of the greatest forms of stress caused to photosynthetic organisms. The penetration of sunlight into water places constraints on the survival and spatial distribution of marine cyanobacteria. Light penetration through the water column is controlled by the amount and kinds of materials that are dissolved and suspended in the water (Batiuk et al., 2000; Kemp et al., 2005). Water turbidity in the Chesapeake Bay is highly variable with highly turbid conditions present in the upper bay. This turbidity creates a low light environment of (10–100 μE m^-2^ s^-1^ within 0.15 and 1 m) (Batiuk et al., 2000). Estuarine *Synechococcus* CB0101 is low light adapted relative to coastal and oceanic *Synechococcus* (**Figure 5**). In the event of upwelling, lower turbidity, or clearing water, CB0101 becomes stressed due to higher light intensities. In order to limit that stress, it is possible that CB0101 is able to induce *relB^2^/relE^1^* TA system, which arrests growth and translation (**Figure 5**).

As photoautotroph, cyanobacteria proliferation is greatly influenced by irradiance. Excessive light can damage cells and cause photo-oxidative cell death. *Prochlorococcus* and *Synechococcus* have different strategies to minimize this photo-oxidative stress (Ting et al., 2002; Franklin et al., 2006). Picocyanobacteria not only need to manage the oxidative stress generated by oxygen reduction, but also produce oxidative oxygen during photosynthetic electron transport (Latifi et al., 2009). ROS are byproducts of aerobic metabolism and potent agents that cause oxidative damage (Latifi et al., 2009). In oxygenic photosynthetic organism such as cyanobacteria, ROS are inevitably generated by photosynthetic electron transport. Phototrophs are able to overcome the damage due to ROS by repairing the photosystem II efficiently (Ohnishi et al., 2005; Latifi et al., 2009). Based on these findings, along with the observation of potential persister state effect of the *relB^2^/relE^1^* system, it seems reasonable to propose that the activation of the *relB^2^/relE^1^* system might redirect the energy normally utilized for growth to aid in repair mechanisms caused by brief amounts of high light intensities. In this model, the *relB^2^/relE^1^* system is envisioned to delay programmed cell death and instead undergo a persister state. With TA systems found within GIs we can predict that they were acquired through horizontal gene transfer from other bacteria, perhaps from freshwater ecotypes where TA systems are common.

### Presence of TA Systems in Broader Picocyanobacteria

Based on a search of *Synechococcus* and *Prochlorococcus* genomes publically available on NCBI (described in Materials and Methods), eight *Synechococcus* strains ranging from estuarine, coastal, and open ocean (CB0205, WH5701, WH8102, WH8103, RS9916, RS9917, KORDI-49, and KORDI-51) with putative hits to at least one pair of TA were identified. Hits to current *Prochlorococcus* genomes were not found. Our results suggest that TA systems in marine *Synechococcus* are probably more widely dispersed than what we thought. Although it is not our focus here to conduct a thorough investigation of the distribution of TAs in marine picocyanobacteria, the diversity and function of TA systems deserves further investigation.

It has been proposed that the absence of TA systems in prokaryotes with small genomes could be due to their relatively simple life style in stable environmental conditions (Pandey and Gerdes, 2005). Makarova et al. (2009) argued that the lack of TAs in small genomes is a consequence of the general “laws” of scaling of differential functional categories of genes with genome size. It has been predicted (with 95% confidence) that no TAs are present in microbial genomes, which contain less than 3,100 genes (Makarova et al., 2009). This is clearly not the case in our study since TAs are present in marine *Synechococcus* strains CB0101, CB0205, WH5701, WH8102, WH8103, RS9916, RS9917, KORDI-49, and KORDI-51, whose genomes contain less than 3,100 genes. Further work is needed to understand the number, type, diversity, evolution and ecological significance of TAs in marine picocyanobacteria. It is clear that TA systems play a major role in regulation of *Synechococcus*.

## Conclusion

Here we described the first TA systems in marine picocyanobacteria. *Synechococcus* strain CB0101, a member of marine *Synechococcus* subcluster 5.2, contains seven chromosomal toxin–antitoxin gene pairs which were expressed in response to five different stressors; nitrogen or phosphate starvation, zinc toxicity, phage infection and photo-oxidative stress. Based on the TA pair *relB^2^/relE^1^* we demonstrated that the transcription of the TA system decreases with the growth and translation under stress conditions, and increases with growth when the adverse event is removed. The up-regulations of toxin genes like *relE^1^* (>10-fold), *doc^1^* and *yoeB^2^* (both >2-fold) were observed when CB0101 was under the Zinc toxicity. Meanwhile, the down-regulations of antitoxin genes such as *relB^2^* and *phd^2^* were observed under the Zinc toxicity. Higher ratios of *relE^1^* vs. *relB^2^* were seen when *Synechococcus* like CB0101 responded to oxidative stress caused by variable light intensities in the Chesapeake Bay. Therefore, the presence of numerous TA systems in the chromosome of CB0101 may contribute to its ability to acclimate to variable conditions in the estuarine ecosystem. The growth regulation of chromosomal type II TA systems may promote marine *Synechococcus* to cope with stressful environments.

The BLAST search on known marine *Synechococcus* and *Prochlorococcus* genomes led to the finding of TA pairs present in eight different marine *Synechococcus* strains isolated from estuarine, coastal, and open ocean environments. Intriguingly, none were found within *Prochlorococcus*. The presence of TA genes in diverse *Synechococcus* spp. suggests that we know very little about the diversity, evolution and ecological functions of TA genes in marine picocyanobacteria. Evaluating TA system-mediated growth regulation of marine picocyanobacteria in response to various environmental changes, i.e., temperature and salinity, is important toward understanding the effect of climate change on ecosystem function.

## Author Contributions

DM performed the experiment and prepared the manuscript. AP contributed to the antibody detection of TA proteins and manuscript discussion and revision. DF helped on manuscript preparation and submission. FC contributed to the experimental design, result discussion, manuscript revision and overall support of this study.

## Conflict of Interest Statement

The authors declare that the research was conducted in the absence of any commercial or financial relationships that could be construed as a potential conflict of interest.
