# Supplementary material for: Toxin-Antitoxin Systems in Estuarine Synechococcus Strain CB0101 and Their Transcriptomic Responses to Environmental Stressors
Source: Front Microbiol. 2017 Jul 6;8:1213. doi: 10.3389/fmicb.2017.01213 (PMC5498466; doi:10.3389/fmicb.2017.01213)
Supplement: Supplementary file 1 [file Table_1.DOCX]

**SUPPLEMENTAL MATERIALS**

**Surviving in a highly variable environment - the novel toxin-antitoxin systems in the estuarine *Synechococcus* strain CB0101**

David Marsan^1^, Allen Place^1^, Daniel Fucich^1^, and Feng Chen^1,^*

^1^Institute of Marine and Environmental Technology, University of Maryland Center for Environmental Science, Baltimore, MD, USA

*correspondence: Feng Chen, Email: [chenf@umces.edu](mailto:chenf@umces.edu), Phone: 410-234-8866

Table S1. Protein description of the TA systems found within CB0101. Toxin represented by bold and anti-toxin by italics. *Bold amino acid sequences highlighted for RelB^2^ and RelE^1^ were used as epitopes for antibody production.

| TA Family | Amino Acid Sequences | PI | Mw |
| --- | --- | --- | --- |
| *yefM^1^* | MGSISASEARRRLFALIDEVRESHQPVEIHGKRGSAVLLAEQDWRAIQETLYLCAIPGMRESILEGLATPVEELSEDAGW | 4.77 | 8891.07 |
| **yoeB^1^** | MSWRVLFTRQAQKDARKLASSSPALKNKAQQLLELLAVDPFQQPPPFEALVGDLQGAYSRRINIQHRLVYAVDREAAVVKVLRLWSHYD | 9.98 | 10215.81 |
| *phd^1^* | messigafeakaqlsrllraveqgehftitvrgrpvadlvphrspssegvaaaiealqafp  rirgvsdadvtsfvaegrr | 4.95 | 14900.04 |
| **doc^1^** | MTLVIDASMALAWVFERQQASDAQRASELLACCGQQAWWVPGLWHLEVANALLVAERRGVIAQDASDLFLVRLSSLPICTDSDAGPEQQ  PRLIALARAHGLSSYDATYLDLAHRLGAALASFDRQLNQAAVAMGVPLT | 6.92 | 9311.45 |
| *vapB^1^* | MTASLPSRVFNNGNSQAVRIPAEFRLGTDQVQISRTPEGDLLIHPCPPQRGQALFNALAGFDADFAERLEQSRAEAQPIQTREGL | 5.21 | 9319.42 |
| **vapC^1^** | MIYLLDTNILIYLIKQRPPEVAERIDQLPGTAQLAMSFITWAELLQGAVGSSRRDAVERQLDHLARQVEVLYPEDSQICRHYAEQATALRRAGTPIGANDLWIACHALAVDATLVTHNLREFTRMSGLSVVDWVQQP | 5.48 | 15422.67 |
| *relB^1^* | MHLLLDTHLLIWAMGSPQRLPNGLADMLEDPGNTPLFSVASLWELVIKQAPNKPDFNVQPALLRRALLECGWQELTITANHALAVADLPPLHRDPFDRLLLAQAKADGLLLITADEQLARYPGPIRWMAPLRPSEES | 5.22 | 15254.73 |
| **vapC^2^** | MLLDTGMRQVNMHEAKTHLSRLVEEAAAGESFLICKAGKPMVRVTSIDQSDAPRPLPRRLGLLEGQCSVPDDFDRFGSEAIADLFEGA | 5.02 | 9614.99 |
| *relB^2^* | MAQV**TARLPDDLTAELDA**VAQQLNRCRADVIRQAIEYYLDDIEDLRAGAASLRDPADPVLDWAEVRDVLLAAD | 4.06 | 8081.04 |
| **relE^1^** | MCCSLRIKRSAAKALAELPNADRLRLVAAIDKLCEVPAAGSALKGEFEGLRRLRVGRYRIVCEWQQQELV**VLVVRVGHRKEVYR** | 9.95 | 9533.32 |
| *mazE^1^* | MTMRYDGEAVVRARLFMSGRSQALRLPARLRLRGPDVEIEPIGDGLWVQPCADPSEGLGDWLERFYADHPPLPVEFLEDRQDQQPQERDWA | 4.64 | 10520.84 |
| **mazF^1^** | MRRTLDTNICSYVLRKRPVQVVERFRQLDRRQLWLSAIVAAELRFGAEKLGSSRFRGSVEAWLSGFELRDWPLAATHHYARLRAQLEAKGTPVGNLDLMIAAHALAEDSVVITNNAREFHRIPGVAVEEWQLD | 9.5 | 15208.44 |
| *phd^2^* | MRTVNVHEAKTHFSRLIDAAHAGETIVVAKGGKPWARLVPLETPAPQRQPGVLAGQLQLPPPEILLEALPEDELRAFEIPLP | 5.86 | 8944.4 |
| **doc^2^** | MSAATAWELATKVRLGKLEIAEPLLSDLPCLLAAQGFELLSVDLRHGLRAGGYPHAHRDPFDRLLVAQAELESLTLVSINAALRDFPCRLLW | 5.84 | 10133.81 |

Table S2. Toxin-antitoxin pairs as identified in the genome of the estuarine *Synechococcus* CB0101 during phage infection by S-CBP1. Toxin represented by bold and anti-toxin by italics.

| TA Pair # | TA Family | Toxin Activity | Viral 30min RNA-Seq | Viral 30min qPCR | Viral 5hr RNA-Seq | Viral 5hr qPCR | Viral 12hr RNA-Seq | Viral 12hr qPCR |
| --- | --- | --- | --- | --- | --- | --- | --- | --- |
| 1 | *yefM^1^* | mRNA interferase or inhibitor of translation initiation | -1.2 | -1.1 | -1.1 | -1.1 | -1.2 | -1.1 |
| 1 | **yoeB***^1^* |  | -1.0 | -1.0 | -2.0 | -1.8 | -1.6 | -1.4 |
| 2 | *phd^1^* | Binds to the 30S ribosomal subunit | -1.8 | -1.9 | -2.0 | -2.0 | -2.9 | -2.7 |
| 2 | **doc***^1^* |  | -1.8 | -1.5 | 1.3 | 1.4 | -1.3 | -1.3 |
| 3 | *vapB^1^* | Cleavage of tRNA | 1.1 | 1.0 | 1.0 | 1.0 | -1.2 | -1.1 |
| 3 | **vapC***^1^* |  | -1.0 | -1.0 | 1.4 | 1.4 | -1.1 | -1.1 |
| 4 | *relB^1^* | Cleavage of tRNA | 1.0 | 1.0 | -1.0 | -1.0 | -2.1 | -2.0 |
| 4 | **vapC***^2^* |  | -1.1 | -1.0 | -1.1 | -1.0 | 1.0 | 1.0 |
| 5 | *relB^2^* | Cleavage of ribosome-bound mRNA | -1.0 | -1.0 | -1.1 | -1.1 | -1.3 | -1.2 |
| 5 | **relE***^1^* |  | 2.5 | 2.6 | -1.3 | -1.2 | 1.7 | 1.7 |
| 6 | *mazE^1^* | Ribosome-independent mRNA cleavage and cleavage of 23S rRNA | -1.1 | -1.1 | -1.5 | -1.4 | -2.3 | -2.2 |
| 6 | **mazF***^1^* |  | -3.2 | -3.1 | 1.3 | 1.2 | -1.1 | -1.3 |
| 7 | *phd^2^* | Binds to the 30S ribosomal subunit | -1.0 | -1.0 | 1.0 | 1.0 | 1.5 | 1.5 |
| 7 | **doc***^2^* |  | -1.4 | -1.3 | -1.4 | -1.5 | -1.3 | -1.3 |

Table S3. Primers developed for qPCR confirmation of RNA-Seq transcript expression. Toxin represented by bold and anti-toxin by italics.

| TA Pair # | TA Family | **Forward Primer** | **Reverse Primer** |
| --- | --- | --- | --- |
| 1 | *yefM^1^* | cgttgagattcacggcaag | tcgctcagttcctccacag |
| 1 | **yoeB^1^** | caagctcgctagctcctcac | tcagtcgtagtggctccaga |
| 2 | *phd^1^* | gagtgagcttttggcttgct | cagataggtggcgtcgtagc |
| 2 | **doc^1^** | acggttactgagggctgttg | cacatctgcatccgaaacac |
| 3 | *vapB^1^* | gtcattgcctagccgtgtgt | gattgctccaaacgttcagc |
| 3 | **vapC^1^** | caggtggaggtgctgtatcc | acccaatccaccacagacag |
| 4 | *relB^1^* | ctggacacacacctgctgat | agttcttgccagccacactc |
| 4 | **vapC^2^** | acccacctctctcgcctagt | tacagaacattggccctcca |
| 5 | *relB^2^* | aatcgatgccgagctgac | ctcagcccaatccagaacag |
| 5 | **relE^1^** | acaagctttgcgaggttcc | gatacacctccttgcgatgc |
| 6 | *mazE^1^* | gatatgacggtgaggctgtg | gagggtgatcggcatagaag |
| 6 | **mazF^1^** | gctctccgggtttgagttg | caatccaactgccactcctc |
| 7 | *phd^2^* | ttccacattcagcaggacag | gttgatcgcgcacgtagat |
| 7 | **doc^2^** | tctgagtgatctgccgtgtc | agcgtgagtgattccagctc |
