# Supplementary figures and images for: Toxin-Antitoxin Systems in Estuarine Synechococcus Strain CB0101 and Their Transcriptomic Responses to Environmental Stressors
Source: Front Microbiol. 2017 Jul 6;8:1213. doi: 10.3389/fmicb.2017.01213 (PMC5498466; doi:10.3389/fmicb.2017.01213)

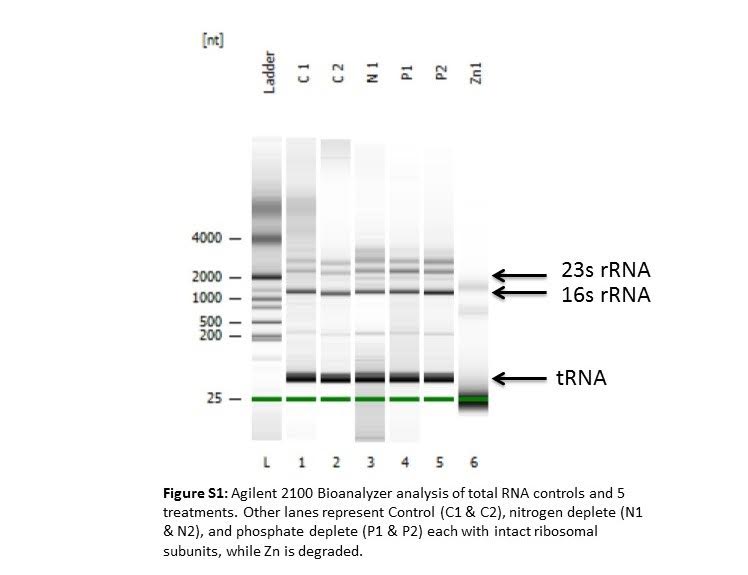

Supplement: Supplementary file 2 [file Image_1.jpeg]
